# Functional Characterization of the Short Neuropeptide F Receptor in the Desert Locust, *Schistocerca gregaria*

**DOI:** 10.1371/journal.pone.0053604

**Published:** 2013-01-04

**Authors:** Senne Dillen, Sven Zels, Heleen Verlinden, Jornt Spit, Pieter Van Wielendaele, Jozef Vanden Broeck

**Affiliations:** Molecular Developmental Physiology and Signal Transduction, Department of Biology, KU Leuven, Leuven, Belgium; University of Rouen, France

## Abstract

Whereas short neuropeptide F (sNPF) has already been reported to stimulate feeding behaviour in a variety of insect species, the opposite effect was observed in the desert locust. In the present study, we cloned a G protein-coupled receptor (GPCR) cDNA from the desert locust, *Schistocerca gregaria.* Cell-based functional analysis of this receptor indicated that it is activated by both known isoforms of *Schgr*-sNPF in a concentration dependent manner, with EC_50_ values in the nanomolar range. This *Schgr*-sNPF receptor constitutes the first functionally characterized peptide GPCR in locusts. The *in vivo* effects of the sNPF signalling pathway on the regulation of feeding in locusts were further studied by knocking down the newly identified *Schgr*-sNPF receptor by means of RNA interference, as well as by means of peptide injection studies. While injection of sNPF caused an inhibitory effect on food uptake in the desert locust, knocking down the corresponding peptide receptor resulted in an increase of total food uptake when compared to control animals. This is the first comprehensive study in which a clearly negative correlation is described between the sNPF signalling pathway and feeding, prompting a reconsideration of the diverse roles of sNPFs in the physiology of insects.

## Introduction

Peptides of the short neuropeptide F (sNPF) family are widely distributed throughout the phylum of Arthropoda. Their occurrence was demonstrated in all arthropod species of which ample sequence data are available. As yet, sNPF has not been observed in non-arthropod species [1]. In most species, multiple sNPF isoforms are derived from a single peptide precursor. In *Drosophila melanogaster*, for example, the *Drome-*sNPF precursor encodes four sNPF isoforms [2], and three putative sNPF peptides were found in the sNPF precursor sequence of *Bombyx mori* [3]. In *Rhodnius prolixus*, however, the *Rhopr*-sNPF pre-pro-peptide precursor only yields one single sNPF-like peptide [4].

In the desert locust, *Schistocerca gregaria*, this situation is confounded by the occurrence of two sNPF-like peptides, as well as a short, truncated form of the *Schgr-*neuropeptide F (*Schgr-*NPF). Both *Schgr*-sNPFs (*Schgr-*sNPF: SNRSPSLRLRFa; *Schgr-*sNPF^4−11^: SPSLRLRFa) were recently identified by means of mass spectrometry and have a widespread occurrence throughout the locust neuroendocrine system, as their presence has been confirmed in the brain, corpora allata, corpora cardiaca, the recurrent and oesophageal nerves, and several ganglia [5], [6]. The sequence of *Schgr*-sNPF^4−11^ is identical to that of sNPF-2 of the honey bee, *Apis mellifera,* which is encoded in the *Apime*-sNPF precursor, along with the longer *Apime*-sNPF-1 (SQRSPSLRLRFa) [7]. The other peptide, truncated NPF (*Schgr*-trNPF: YSQVARPRFa), was at the time of its discovery grouped under the NPF-like peptides, but was characterized by its remarkably short length. Whereas the length of other known NPF-like peptides ranged between 36 and 39 amino acids, the *Schgr-*trNPF was only nine amino acids in length [8]. Recent publication of a *S. gregaria* EST database revealed that this *Schgr-*trNPF corresponds to the carboxyterminal fragment of a full-length *Schgr-*NPF, encoded by a contig, built from partial transcripts present in the central nervous system of *S. gregaria* [9]. A similar situation was found in the corn earworm, *Helicoverpa zea*, where more than a decade after its initial discovery, Midgut Peptide I was shown to correspond to the carboxyterminal fragment of the full-length *Helze*-NPF [10].

Although the peptides of the sNPF family appear to be implicated in a wide range of processes, including locomotor activity [11] and circadian rhythms [12], their main function appears to lie in the regulation of feeding behaviour. In *Drosophila melanogaster*, overexpression of *Drome-*sNPF promoted food intake in both larvae and adult flies, and yielded larger and heavier flies. Correspondingly, knocking down the *Drome-*sNPF precursor resulted in the opposite phenotype. However, it should be pointed out that the effects of the gain-of-function and loss-of-function sNPF phenotypes were only observed in larvae in the feeding stage of development and that wandering larvae showed no such response [13]. Similar effects to those of sNPF overexpression were observed when the *Drosophila* sNPF receptor, *Drome-*sNPFR1, was overexpressed [14]. In several insects, the expression of the sNPF receptor (sNPFR) was also shown to be linked to food search behaviour, since increased transcript levels were found in both starved fruit flies [15], cockroaches [16], and foraging honeybees [17]. Furthermore, injection of *Bommo-*sNPF-2 significantly reduced the latency to feeding of starved larvae of the silk moth, *Bombyx mori* [18]; while injection of sNPF into the American cockroach, *Periplaneta americana*, resulted in a general decrease of digestive activity, suggesting a correlation with the starved state [16].

In other insect species, the sNPF signalling pathway did not generate stimulatory effects on feeding behaviour, but either negatively regulated feeding or was correlated with the non-feeding state. While injection of *Bommo-*sNPF-2 resulted in a decreased latency to feeding [18], a recent study by Nagata *et al*. showed that the levels of *Bommo-*sNPF-1, *Bommo-*sNPF-2, and the *Bommo-*sNPF receptor decreased upon starvation, when animals were more inclined to engage in food searching behaviour [19]. In the red imported fire ant, *Solenopsis invicta*, transcript levels of the sNPF receptor where found to be downregulated in starved queens compared to their non-starved congeners [20]. In the Colorado potato beetle, *Leptinotarsa decemlineata*, the *Lepde*-sNPF peptide could not be detected in diapausing animals [21]. These findings show that, in these animals, there is no obvious positive correlation between sNPF and food search behaviour. Further indications for a possible negative influence of sNPF on feeding came from experiments in the yellow fever mosquito, *Aedes aegypti*, in which the hemolymph titre of Head Peptide I (*Aedae-*HP-I), an *Aedae-*sNPF [22], increased fivefold after a replete blood meal. Furthermore, injection of *Aedae-*HP-I in female mosquitoes rendered them refractory to host cues and significantly inhibited host-seeking behaviour for up to five hours [23].

In this paper, we describe the characterization of a sNPF receptor from the desert locust, *Schistocerca gregaria*, and discuss its role in the regulation of food uptake. We show that this receptor is activated by both *Schgr*-sNPFs. Furthermore, we also show that *in vivo* injection of sNPF results in a significant reduction of food uptake, while knocking down the receptor transcript levels by means of RNA interference (RNAi) increases food uptake.

## Materials and Methods

### 1. Peptides

The peptides used in this study (supplied by GL Biochem Ltd) were purified by means of reverse-phase HPLC. The purity of the obtained fractions was then verified using a MALDI TOF-TOF Ultraflex II mass spectrometer (Brucker Daltonics) and their concentration was determined on a Qubit® Fluorometer (Invitrogen), using the Quant-It™ assay (Invitrogen). They were then lyophilized and diluted to the desired concentrations.

### 2. Cloning of the *Schgr-*sNPF Receptor cDNA

The sequence encoding the *Schgr-*sNPFR was amplified by performing PCR on cDNA derived from the brains of adult locusts. The specific oligonucleotide primers used were: Fw 5′-ACCGCAGCAGCAGCAGCACTTGT-3′ and Rv 5′-CCACACGTGCGGAAAGGCATCA-3′ (Sigma-Aldrich) and the following cycling programme was used: 95°C for 120 s, followed by 30 cycles of 95°C for 30 s, 70°C for 30 s and 68°C for 60 s. After these cycles, the programme was completed with a final elongation step of 68°C for 120 s and ended at 4°C. The PCR product was run on a 1% agarose gel, from which it was purified using the GenElute™ Gel Extraction Kit (Sigma-Aldrich). Amplicons were then cloned in a pcDNA3.1D/V5-His-TOPO® expression vector (Invitrogen), which were transformed into One Shot TOP10 chemically competent *Escherichia coli* cells (Invitrogen). These were plated on LB agar plates (35 g/l, Sigma-Aldrich) with ampicillin (10 mg/ml, Invitrogen) and grown overnight at 37°C. Colonies were collected and transferred to LB medium (25 g/l, Sigma-Aldrich) with ampicillin (10 mg/ml, Invitrogen) and again grown overnight at 37°C. Plasmids were extracted using the GenElute™ HP Plasmid Miniprep kit (Sigma Aldrich) and the insert sequences were verified on a ABI PRISM 3130 Genetic Analyzer (Applied Biosystems) using the ABI PRISM BigDye Terminator Ready Reaction Cycle Sequencing Kit (Applied Biosystems).

### 3. Cell Culture and Transfections

Three cell lines were used in this study. General receptor studies were performed in Chinese Hamster Ovary (CHO)-WTA11 cells, a clone of CHO cells stably coexpressing the promiscuous G_α16_ and apoaequorin. To determine the mode of downstream signalling, we used either CHO-PAM28 cells, a clone of CHO cells expressing apoaequorin but not the promiscuous G_α16_, or Human Embryonic Kidney (HEK) 293T cells, expressing neither G_α16_ nor apoaequorin. All cell lines used in this study were provided by Prof. Dr. M. Parmentier and Dr. M. Dethreux (Université Libre de Bruxelles) and Euroscreen S.A., Belgium.

The cells were cultured in monolayer, in Dulbecco’s Modified Eagle Medium Nutrient Mixture F12-Ham (DMEM/F12, Invitrogen) containing 10% fetal calf serum (Invitrogen), 100 IU/ml penicillin/streptomycin (Invitrogen). For the CHO-WTA11 cells, 250 µg/ml Zeocin™ (Invitrogen) was added to the medium, while to the medium of CHO-PAM28 cells, 5 µg/ml Puromycin (Invitrogen) was added. The cells were grown at 37°C, with a constant supply of 5% CO_2_ and split every 3 days.

Transfections with either *Schgr-*sNPFR/pcDNA3.1D or empty pcDNA3.1D vector were performed in T75 flasks at *ca.* 60% confluency. Transfection reagent was prepared by combining 2,5 ml of DMEM/F12, 5 µg of the plasmid DNA and 12.5 µl of PLUS™ Reagent (Invitrogen). After storing the medium at room temperature for 5 minutes, 30 µl Lipofectamine™ LTX (Invitrogen) was added and the transfection medium was added dropwise into 3 ml of culture medium after 30 minutes incubation at room temperature. HEK 293T cells were cotransfected with a reporter construct, consisting of a *luciferase* gene situated downstream of a cyclic AMP (cAMP) response element (CRE) and promoter.

### 4. Bioluminescent Assays

In the calcium-assays, CHO-WTA11 or CHO-PAM28 cells expressing the *Schgr-*sNPFR were detached two days after transfection, counted using the NucleoCounter® NC-100™ (Chemometec), and resuspended in DMEM/BSA (DMEM/F12, 10 mM HEPES, 0.1% bovine serum albumin) at a concentration of 5×10^5^ cells/ml. Cells where then shielded from light and incubated for 4 h with 5 µM coelenterazine h (Invitrogen), allowing the aequorin holoenzyme to be reconstituted.

After incubation, the cell suspension was injected in wells containing a peptide solution, dissolved in 50 µl BSA medium. After 30 s, 50 µl of 0.1% Triton X-100 was injected, lysing the cells and thus serving as an internal reference. Wells containing no peptide but DMEM/BSA were used as a negative control while wells containing 1 µM ATP were used as a positive control. Light emission was monitored using a Mithras LB 940 Microplate Reader (Berthold technologies).

HEK 293T cells, cotransfected with the *Schgr-*sNPFR/pcDNA3.1 vector and the CRE-*luciferase* construct, were detached two days after transfection, centrifuged and resuspended to a concentration of 10^6^ cells/ml in DMEM/F12 containing 200 µM 3-isobutyl-1-methylxanthine (IBMX; Sigma-Aldrich). Fifty µl of this suspension was introduced into in each well of a 96-well-plate. When studying stimulatory effects, peptides were dissolved in DMEM/F12 containing 200 µM IBMX. When studying inhibitory effects, these peptides were dissoved in DMEM/F12 supplemented with 200 µM IBMX and 20 µM NKH 477 (a water-soluble analogue of forskolin; Sigma-Aldrich). Subsequently, 50 µl of the peptide solution was introduced into the wells containing the cell suspension and the plate was incubated for 3 hours at 37°C. Visualization of the luciferase enzymatic activity was accomplished by the addition of 100 µl SteadyLite Plus™ (Perkin Elmer), after which the plate was shielded from light and gently shaken for 15 minutes. Light emission, resulting from the luciferase activity, was measured for 5 s/well using the Mithras LB 940 Microplate Reader.

Data generated in these experiments were analysed using GraphPad Prism 5 (GraphPad Software).

### 5. Animal Rearing Conditions

The desert locusts used in this study were all gregarious adult animals. They were reared under crowded conditions at a constant temperature (32±1°C) and photoperiod (14 h). The locusts were kept at high density (>200 locusts per cage) and fed daily with cabbage and dry oat flakes. In an effort to normalize feeding responses, locusts used in feeding experiments were placed in separate cages and placed on a controlled dietary regimen one week prior to the assay. Locusts were fed for two hours each day (9 h–10 h, 16 h–17 h).

### 6. Receptor Transcript Distribution

Tissues were dissected under a binocular microscope, collected in tubes containing MagNa Lyser Green Beads (Roche) and immediately frozen in liquid nitrogen. Prior to RNA extraction, the tissues were homogenized using the MagNa Lyser instrument. Total RNA was then isolated using the RNeasy® Lipid Tissue Mini Kit (Qiagen), in combination with a DNase digestion of the purified nucleic acids (RNase-free DNase Set, Qiagen). The resulting total RNA was reverse transcribed to cDNA with the Superscript™ III Reverse Transcriptase (Invitrogen), using 1 µg total RNA and random hexamer primers. The resulting cDNA was diluted tenfold and used as template in quantitative (realtime) reverse transcription PCR (qRT-PCR).

Primer pairs were designed by means of the Primer Express® software (Applied Biosystems; table 1) and subjected to melting curve analysis to verify their specificity and efficiency in amplification. Furthermore, amplification products of PCR reactions were analysed by means of electrophoresis on a 1% agarose gel. Visualization of the PCR products showed a single band of the expected size. Sequencing of these PCR products further verified the specificity of the qRT-PCR amplification.

**Table 1:** Primer sequences for the qRT-PCR assays.

	Forward Primer (5′-3′)	Reverse Primer (5′-3′)
*Schgr-*sNPFR	GCGACGCTCCTGTAGATGTTAA	GTATGTGGCGGATTCGCTAGA
EF1α	GATGCTCCAGGCCACAGAGA	TGCACAGTCGGCCTGTGAT
RP49	CGCTACAAGAAGCTTAAGAGGTCAT	CCTACGGCGCACTCTGTTG

Prior to the assay, several reference genes were analysed using the geNorm software [24], revealing ribosomal protein 49 (RP49) and elongation factor 1α (EF1α) as the most stably expressed pair over the sample set. For the qRT-PCR, we used Fast SYBR® Green Master Mix (Applied Biosystems), as per manufacturer’s instruction, and the StepOnePlus™ Real-Time PCR system (Applied Biosystems). The Fast SYBR® Green Master Mix contains the fluorescent ROX™, which is used as a passive reference. All samples were measured in duplicate and all plates contained a *no template control* for all three primer pairs to check for possible contaminations in the master mix. The following program was used: 95°C for 10 minutes, followed by 40 cycles of 95°C for 3 s and 60°C for 30 s. Data were analysed using the ΔΔC_T_ method, in which the cycle threshold (C_T_) value for the gene of interest (GOI) is normalized to the C_T_ of one or more reference genes (ΔC_T_) and to the C_T GOI_ of a calibrator sample (ΔΔC_T_), included on each plate [25].

### 7. Peptide Injection Studies

At 9 AM, 2 µl of a 100 µg/ml peptide solution (experimental condition) or saline solution (negative control) was injected dorsally between the first and second abdominal segments using a 710 RN injector (Hamilton). Pieces of cabbage were weighed in advance and presented to the locusts for one hour, after which all animals had stopped feeding. In parallel, similar pieces of cabbage were put in empty cages during the period of the experiment. The relative weight loss of these pieces was used to estimate the effect of evaporation All cabbage leafs were weighed after the feeding assay, total food uptake was calculated and corrected for the weight that was lost by evaporation. The results were analysed using GraphPad Prism 5 (GraphPad Software).

### 8. Receptor knockdown

T7 promoter sites were incorporated on either side of a 352 bp fragment of the *Schgr-*sNPFR sequence. This was done by performing PCR, using the following primers: *Schgr-*sNPFR-T7-Fw 5′-TAATACGACTCACTATAGGGAGAGCAGATACGCCTTCCAGAAA-3′ and *Schgr-*sNPFR-T7-Rv 5′-TAATACGACTCACTATAGGGAGACTGCCCAGGAAGGTGTAGAG-3′. The amplicon was then subcloned and analysed as described above (2.2).

Double stranded RNA (dsRNA) was generated using the MEGAscript® RNAi Kit (Ambion) and its length was checked on a 1% agarose gel. Knockdown efficiency was assayed at RNA level by qRT-PCR. Animals were injected with either 2 µl 100 ng/µl dsRNA or 2 µl saline solution. For both conditions, 25 animals were placed on the dietary regimen previously described while another 25 animals were starved. Five days after injection, at the time of the first daily feeding session, the animals were given pieces of cabbage of which the weight was previously determined and were allowed to eat for one hour. Once again, total food uptake was calculated and corrected for weight lost by evaporation. Results were analysed using GraphPad Prism 5 (GraphPad Software).

## Results

### 1. *Schgr-*sNPF Receptor Sequence

**Figure 1:**
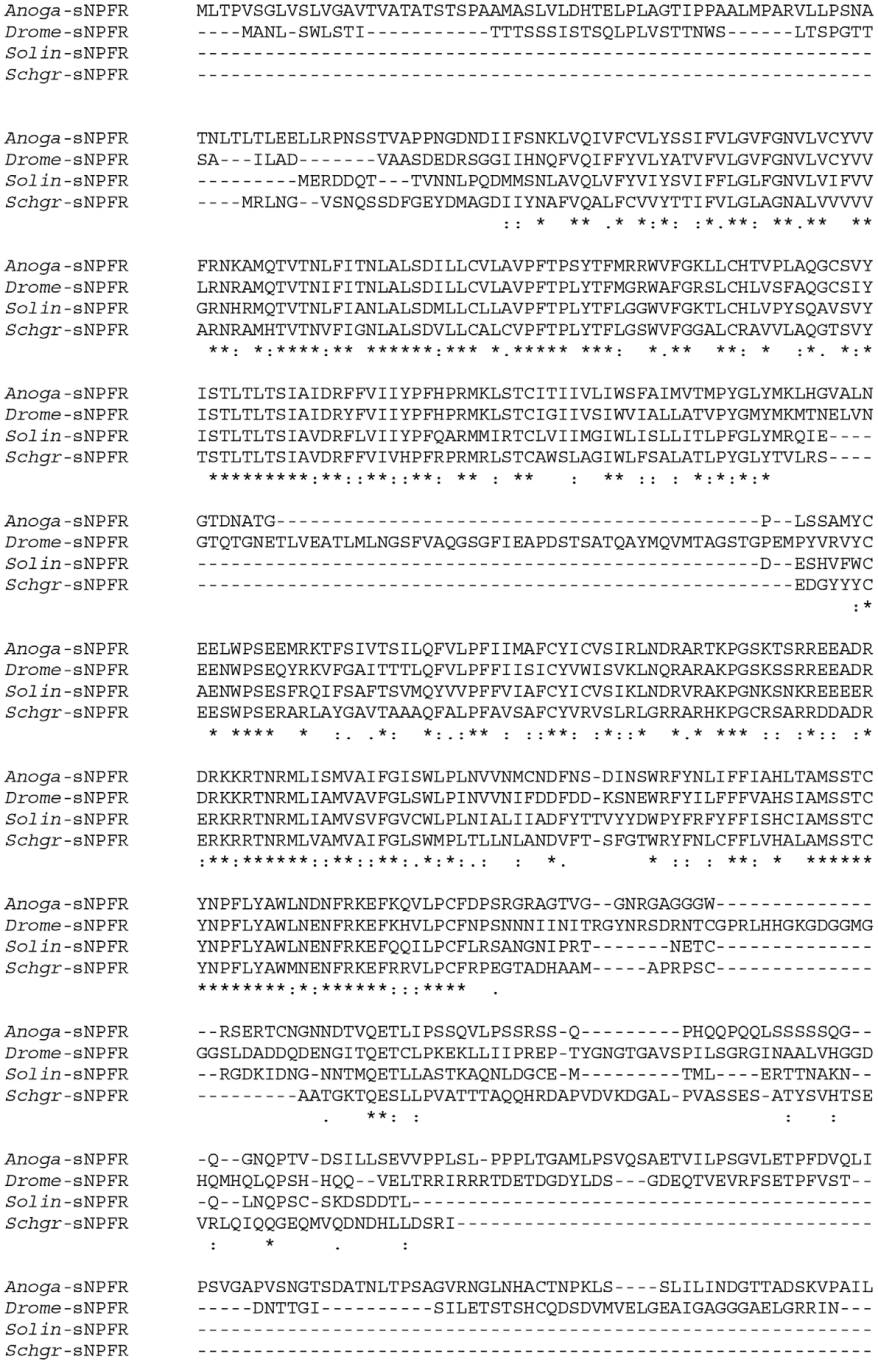
Analysis of sNPF receptor sequence conservation. Multiple sequence alignment of amino acid sequences of insect sNPF receptors, performed with Clustal Omega software, available from the European Bioinformatics Institute. Default parameters were used. *Anoga*: *Anopheles gambiae* (ABD96049.1), *Drome*: *Drosophila melanogaster* (AAF49074.2), *Solin*: *Solenopsis invicta* (AAY88918.1), *Schgr*: *Schistocerca gregaria* (JX855828). ‘*’ identities, ‘:’ conservative substitutions, ‘.’ semi-conservative substitutions.

Based on the known sequences of the short neuropeptide F receptors in other insect species, we identified the *Schgr-*sNPF receptor in the recently published *Schistocerca gregaria* EST database [9]. The receptor cDNA sequence was completed by sequencing the EST-clone in both 5′ and 3′ directions. In addition, the receptor sequence was confirmed by PCR cloning from locust brain cDNA and DNA sequencing, and uploaded to Genbank (accession number JX855828; figure 1). The presence of seven alpha-helical transmembrane segments was predicted using TMHMM 2.0 software (Center for Biological Sequence Analysis, Technical University of Denmark), followed by a stop codon and poly(A) sequence, indicating that the full coding sequence is represented (figure S1).

### 2. Receptor Ligands and Cell-based Functional Receptor Analysis

As putative ligands for the cloned *Schistocerca gregaria* sNPF receptor, *Schgr-*sNPF (SNRSPSLRLRFa), *Schgr-*sNPF^4−11^ (SPSLRLRFa) and *Schgr-*trNPF (YSQVARPRFa) were tested. Despite the fact that the latter does not belong to the short neuropeptide F family, it shows C-terminal sequence similarity to these peptides, prompting its inclusion in this study. For each of these peptides, the dose-dependent activation of the *Schgr-*sNPFR was studied in CHO-WTA11 cells and EC_50_ values were calculated. No dose-dependent response was observed when the receptor was incubated with *Schgr-*trNPF, indicating that the receptor is specific to members of the sNPF family, nor was any response observed in the cells transfected with empty pcDNA3.1D vector.

**Figure 2:**
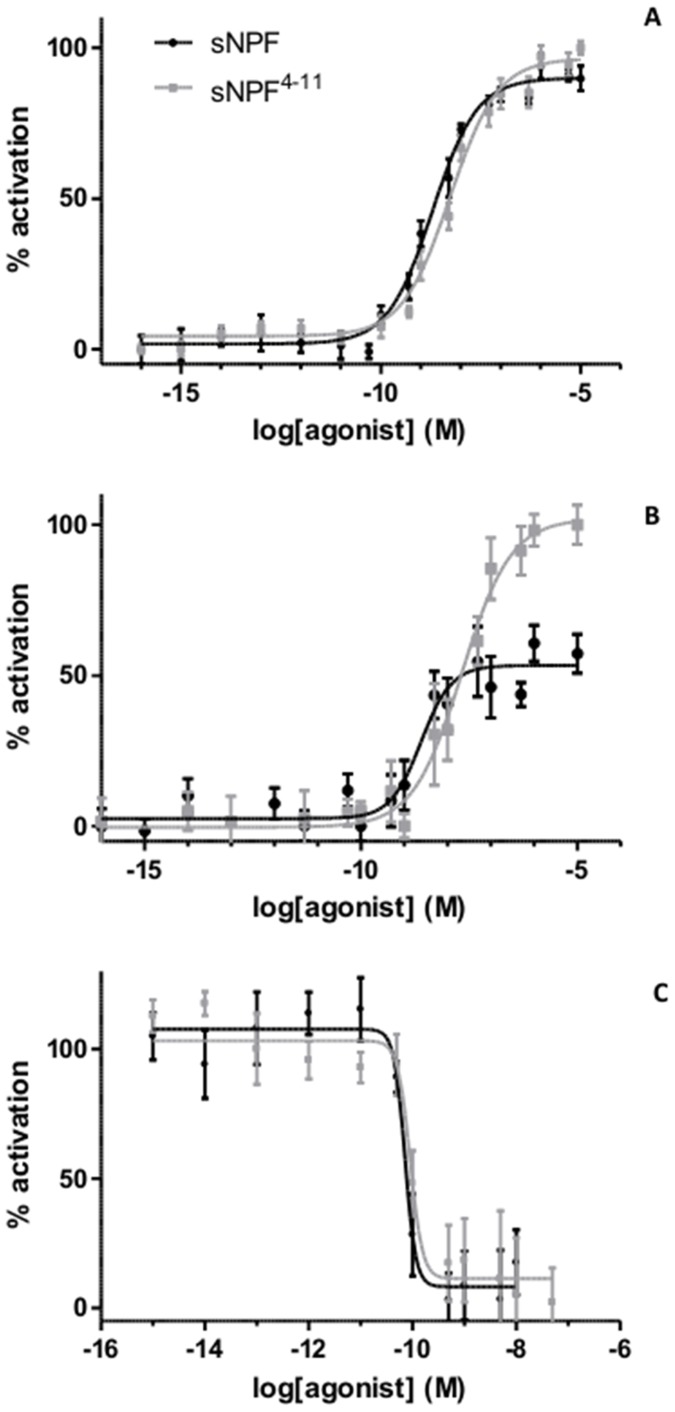
Dose-response curves for bioluminescence induced by *Schgr-*sNPF and *Schgr-*sNPF^4–11^ in Schgr-sNPFR-expressing cells. A. Aequorin bioluminescence induced in CHO-WTA11-*Schgr-*sNPFR cells, stably expressing the promiscuous G_α16_ subunit. Data represent the average ± SEM of three independent measurements performed in duplicate and given in percentage of the maximum response. The zero response level corresponds to treatment with BSA solution. B. Aequorin bioluminescence induced in CHO-PAM28-*Schgr-*sNPFR cells. Data represent the average ± SEM of three independent measurements performed in duplicate and given in percentage of the maximum response. The zero response level corresponds to treatment with BSA solution. C. Luciferase bioluminescence induced in HEK-293T-*Schgr-*sNPFR cells. Data represent the average ± SEM of three independent measurements performed in duplicate and given in percentage of the maximum response. The 100% response level corresponds to treatment with DMEM/F12 containing 200 µM IBMX and 20 µl NKH477 (a water-soluble analogue of forskolin).

Both *Schgr-*sNPF peptides were shown to induce activity of the receptor in a dose-dependent way (figure 2A). For *Schgr-*sNPF, an EC_50_ value of 2.05±0.63 nM was calculated (95% confidence interval). The shorter peptide variant, *Schgr-*sNPF^4−11^, showed a slightly higher EC_50_ value of 5.56±1.8 nM.

These two peptides were then further studied with respect to their mode of intracellular signalling. Cell based assays were performed using a CHO-PAM28 cell line, expressing apoaequorin, allowing the detection of increases in the intracellular concentration of calcium ions. Stimulation of the *Schgr-*sNPFR expressing cells with *Schgr*-sNPF or *Schgr*-sNPF^4−11^ resulted in a dose-dependent increase of aequorin bioluminescence. However, the maximum responses obtained with *Schgr*-sNPF^4−11^ where markedly higher than those obtained with *Schgr*-sNPF. The calculated EC_50_ values were both in the nanomolar range. EC_50_ values for *Schgr*-sNPF (EC_50_ = 3.28±2.20 nM) and *Schgr*-sNPF^4−11^ (EC_50_ = 32.2±18.2 nM) were calculated (figure 2B).

The possible effect of receptor activation on intracellular cyclic AMP (cAMP) production was analysed in HEK 293T cells that were cotransfected with a CRE-*luciferase* reporter construct. Inhibition of the reporter expression was highly effective for both *Schgr-*sNPF and *Schgr-*sNPF^4−11^ and IC_50_ values were obtained in the picomolar range (sNPF: 76.3±26.1 pM; sNPF^4−11^: 95.5±32.3 pM; figure 2C).

### 3. Receptor Transcript Distribution and Dependency of Nutritional State

**Figure 3:**
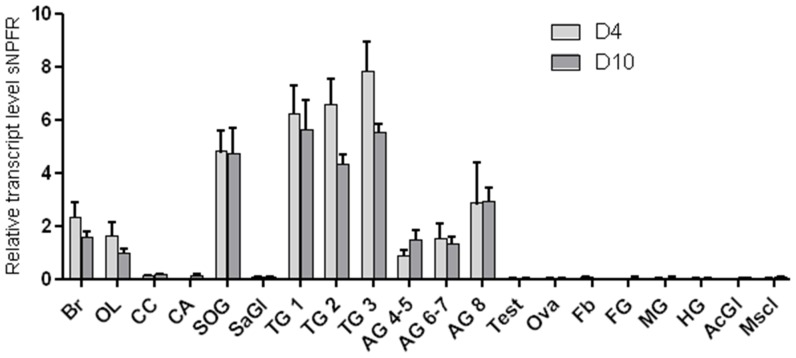
Relative levels of the sNPF receptor transcript four and ten days after adult eclosion. Expression of the sNPF receptor transcript seems limited to neural tissue. Br: brain; OL; optic lobes; CC: corpora cardiac; CA: corpora allata; SOG: suboesophageal ganglion; SaGl: salivary gland; TG1: prothoracic ganglion; TG2: metathoracic ganglion: TG3: metathoracic ganglion; AG4–5: abdominal ganglia 4 and 5; AG6–7: abdominal ganglia 6 and 7; AG8: abdominal ganglion 8; Test: testes; Ova: ovaries; Fb: fatbody; FG: foregut; MG; midgut; HG: hindgut; AcGm: male accessory gland; Mscl: flight muscle. Data represent the average ± SEM (n = 6).

We used quantitative reverse transcription PCR to determine the level of *Schgr-*sNPFR transcript in a wide range of tissues. Both time points tested, either four or ten days after adult eclosion, provided a very similar distribution pattern and no significant temporal differences could be observed. The *Schgr-*sNPFR transcript was detected throughout the central nervous system, with the highest transcript levels found in the suboesophageal ganglion and the thoracic ganglia. Its presence was not observed in flight muscle or tissues of the digestive or reproductive system (figure 3).

**Figure 4:**
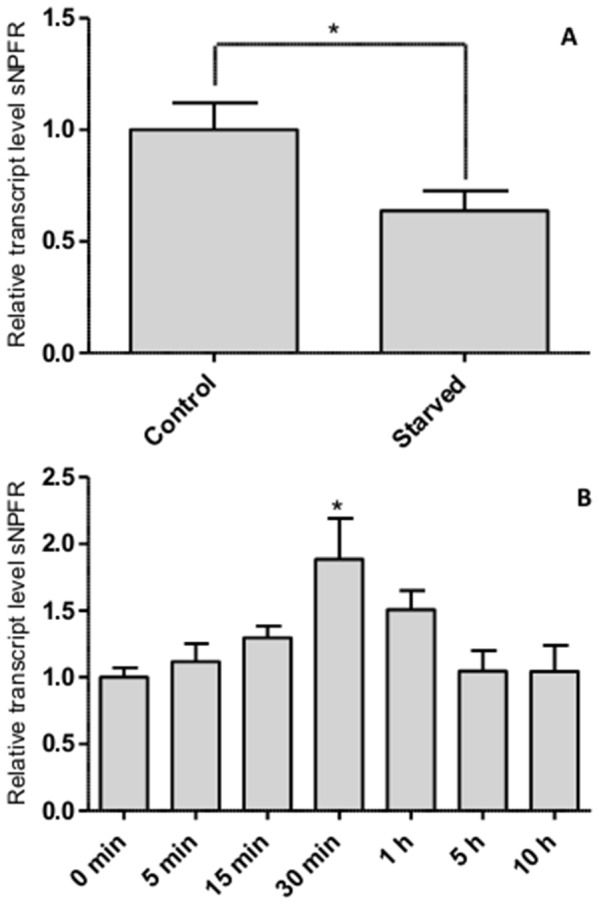
Relative levels of the sNPF receptor transcript upon starvation and after feeding. A. Expression of the *Schgr-*sNPFR transcript in the brain of adult locusts is significantly lower after five days of starvation. Data represent the average ± SEM (n = 10). Results were analysed using Student’s t-test. * p<0.05. B. Expression of the *Schgr-*sNPFR transcript in the brain of male adult locusts peaks 30 minutes after feeding, after which it returns to a baseline level. Data represent average ± SEM (n = 6). Results were analysed using ANOVA in combination with Tukey’s Multiple Comparison Test. * p<0.05.

Significantly lower levels of the *Schgr-*sNPF receptor transcript were detected in the brains of adult locusts after five days of starvation, compared to animals fed *ad libitum* (figure 4A). Furthermore, additional studies showed that the transcript levels of *Schgr-*sNPF receptor peak 30 minutes after the locusts were allowed to feed, after which they returned to a baseline level (figure 4B). Neither feeding, nor starvation, significantly affected receptor transcript levels in the suboesophageal or thoracic ganglia.

### 4. Feeding Assays

**Figure 5:**
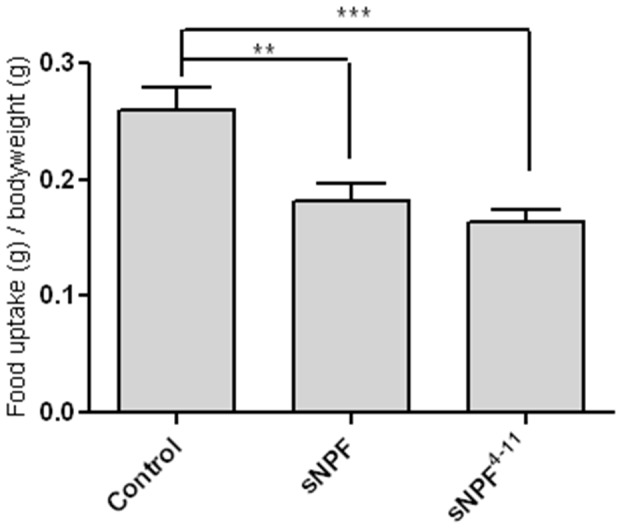
Injection of 200 pg *Schgr-*sNPF or *Schgr-*sNPF^4–11^ in adult locusts and its effect on food uptake, normalized to the body mass of the locusts. A significant decrease in food uptake is observed for both sNPF and sNPF^4−11^. Data represent mean values ± SEM (n = 20). Results were analysed using ANOVA in combination with Tukey’s Multiple Comparison Test. ** p<0.01; *** p<0.001.

**Figure 6:**
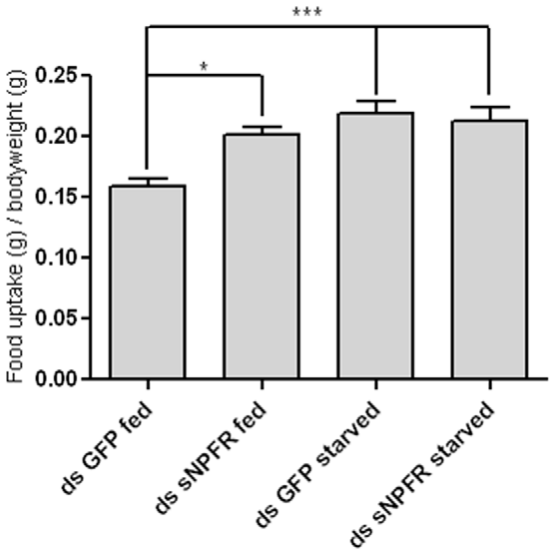
RNAi mediated knockdown of the mRNA encoding the *Schgr-*sNPF receptor and its effect on food uptake. Starved control animals show a higher food uptake than fed controls. Knockdown of the sNPFR transcript mimics the starved state in fed animals. Data represent mean values ± SEM (n = 20). Results were analysed using ANOVA in combination with Tukey’s Multiple Comparison Test. * p<0.05, *** p<0.001.

To further assess the possible *in vivo* function of sNPF signalling in *Schistocerca gregaria*, a series of feeding assays was performed. First, the effects on feeding behaviour of adult locusts were studied by *in vivo* injections of the receptor-agonists. When injected, *Schgr-*sNPF and *Schgr-*sNPF^4−11^ resulted in a reduction of food uptake of respectively 30 and 37%, compared to locusts injected with an equal volume of saline (figure 5). The effect of a reduced activity of the sNPF signalling pathway was studied by knocking down the mRNA encoding the *Schgr-*sNPF receptor. By means of RNAi, a reduction of over 85% of the *Schgr-*sNPFR specific mRNA could be obtained (figure S2). The study showed an augmented food uptake in starved versus fed control animals. Silencing sNPFR expression by means of RNAi resulted in a 24% increase of food uptake in fed animals, while no significant effects were observed in starved locusts (figure 6).

## Discussion

For a given neuropeptide, the identification and characterization of its cognate receptor represents an important step towards the elucidation of its mode of action. All currently identified arthropod sNPF receptors belong to the superfamily of rhodopsin-like G protein-coupled receptors (GPCR), which contain seven transmembrane (serpentine) segments, an extracellular aminoterminal segment and an intracellular carboxyterminal tail [20], [26]–[32]. In the *Schgr-*sNPF receptor amino acid sequence, deduced from its cDNA sequence, seven regions were indeed predicted to encode such transmembrane segments. The receptor encoding sequence is found between a start codon and a termination codon, while a poly(A) tail is present more downstream of the latter. The cloned sequence therefore seems to include the entire open reading frame of the *Schistocerca gregaria* sNPF G protein-coupled receptor (figure S1). Sequence comparison indicates that this receptor resembles other sNPF receptors in insects (figure 1).

Cell based functional studies indicated that the newly identified *Schgr-*GPCR is activated by locust peptides of the sNPF family, which yielded EC_50_ values in the nanomolar range in cells that contain the promiscuous G protein subunit G_α16_ (figure 2A). However, this receptor was not responsive to *Schgr-*trNPF, a peptide belonging to the NPF family. The downstream signalling, generated by the *Schgr-*sNPF receptor, was further studied in CHO-PAM28 and HEK 293T cells. A clear dose-dependent intracellular release of calcium was observed in CHO-PAM28 cells (figure 2B). When the calcium responses of stimulation by both peptides are compared, the maximum response of *Schgr-*sNPF is markedly lower than that of Schgr-sNPF^4−11^, indicating that *Schgr-*sNPF acts as a partial agonist of *Schgr*-sNPFR, with respect to this calcium response. The observed difference in agonist efficiency could point at the existence of an agonist-dependent difference in the activated receptor conformation. It is known that partial agonist behaviour is dependent on receptor density and G protein coupling efficiency, both of which may depend on the cell line that is used [33], [34]. Since this analysis was performed in CHO cells, the results may not necessarily accurately reflect the situation *in vivo*. Nevertheless, it is possible that agonist-dependent, receptor-mediated, differences in downstream coupling behaviour may contribute to the physiological fine-tuning by naturally occurring variants of a given peptide family, as previously suggested by Poels *et al* [35].

In addition, the possible effects of receptor stimulation on the intracellular cAMP levels were studied in HEK 293T cells cotransfected with a CRE-*luciferase* reporter construct. In these assays, luciferase bioluminescence dropped when the cells were challenged with low concentrations of agonist, suggesting a negative coupling of the activated sNPF receptor to adenylyl cyclase (figure 2C). Similar cellular activities were previously demonstrated for the sNPF receptor of *Anopheles gambiae,* where IC_50_ values ranged between 3 to 5 nM [27]. In the present study, IC_50_ values were obtained in the picomolar range, which indicates that this *Schgr*-sNPF-induced signalling pathway is already activated at very low agonist concentrations. While a neuromodulatory role of sNPF was previously demonstrated in *Drosophila melanogaster* [15], the high sensitivity as observed for *Schgr*-sNPFR might also allow its ligand(s) to act as a neuroendrocrine factor.

The distribution of the *Schgr-*sNPF receptor transcript mainly appeared to be limited to the nervous system (figure 3). This sNPFR tissue distribution in locusts contrasts with some previous studies in other insects, where sNPFR expression was observed in a number of peripheral tissues, such as the gut, fat body, and ovaries of the imported fire ant, *Solenopsis invicta*, [20], [36] and *Drosophila melanogaster* [26], in addition to the nervous system. Moreover, the *Schgr-*sNPF receptor transcript levels appear to be dependent on the nutritional state of the locusts. A period of starvation of 5 days resulted in a significant decrease of receptor transcript levels (figure 4A). A similar effect was reported in *Solenopsis invicta* [20] and *Bombyx mori* [19]. In both studies, the authors linked these results to a hypothetical increase in feeding response, as it seemed a valid assumption that starved animals would more readily engage in feeding behaviour.

In the present study, the possible *in vivo* role of the ligand-receptor couple, sNPF/sNPFR, in feeding was further tested by the performance of a series of peptide and dsRNA injection experiments. These revealed that the *Schgr-*sNPF signalling pathway plays an inhibitory role in the regulation of locust feeding. Injection of either *Schgr-*sNPF or *Schgr-*sNPF^4−11^, resulted in a significant decrease of food uptake (figure 5). On the other hand, RNAi mediated knockdown of the *Schgr-*sNPF receptor transcript levels resulted in an increase of food uptake in fed adult locusts (figure 6). It is interesting to note that similar effects were also obtained for the CRF-like diuretic hormone (CRF-DH) of *Schistocerca gregaria* [37]. At present, it is not known whether sNPF and CRF-DH peptides may functionally interact in this *in vivo* process.

In *Aedes aegypti*, studies have shown that the *Aedae-*HP-I, an *Aedes* sNPF, was transiently upregulated after feeding [23]. Correspondingly, the current study also shows a peak of *Schgr-*sNPF receptor transcript levels after feeding (figure 4B). These observations suggest that in both *Aedes aegypti* and *Schistocerca gregaria*, sNPF may be involved in post-feeding signalling, attenuation of food uptake and may act as a satiety signal.

*Schgr-*sNPF receptor transcript levels are higher in fed than in starved animals (figure 4A). This means that when the *Schgr-*sNPF receptor transcript levels of fed animals are reduced by means of RNAi, they probably mimic those of their starved congeners. The effect of such a knockdown can be seen in figure 6, where we show that silencing the *Schgr-*sNPFR transcript increases food uptake to the level observed in starved locusts.

Although these studies show a clear link between sNPF signalling and feeding, the sNPF signalling pathway is probably not the only (neuro)endocrine link between nutritional state and feeding behaviour. In insects, feeding behaviour is likely to be regulated by a broad variety of neuropeptides, as discussed in recent studies [18], [37]–[41] or as reviewed by Spit *et al*. [42].

In conclusion, we show a link between feeding state, sNPF signalling, and food uptake. While feeding increases the expression of *Schgr-*sNPFR, an increased activity of the sNPF signalling pathway attenuates food uptake. This negative feedback control loop, consisting of feeding-induced inhibition of food uptake, provides an interesting mechanism allowing the control of feeding behaviour to be adjusted to the nutritional state of the locust.

## Supporting Information

Figure S1 **Nucleotide sequence and corresponding amino acid sequence of the short neuropeptide F receptor transcript of** ***Schistocerca gregaria*** **.** The open reading frame is printed in black, 5′- and 3′-untranslated regions are printed in grey, sequences predicted as transmembrane segments are underlined, primer sequences used in qRT-PCR assays are highlighted, and the region corresponding to the dsRNA used in RNAi studies is printed in bold.
(TIF)

Figure S2 **Relative levels of the sNPF receptor transcript upon** ***Schgr-*** **sNPFR knockdown.** Locusts were injected with 200 ng dsRNA corresponding to either GFP or sNPFR. Injection of sNPFR dsRNA resulted in a 85% reduction in transcript levels five days after injection. Data represent mean values ± SEM (n = 8). Results were analysed using Student’s t test. *** p<0.001.
(TIF)
