# Supplementary figures and images for: Functional Characterization of the Short Neuropeptide F Receptor in the Desert Locust, Schistocerca gregaria
Source: PLoS One. 2013 Jan 4;8(1):e53604. doi: 10.1371/journal.pone.0053604 (PMC3537624; doi:10.1371/journal.pone.0053604)

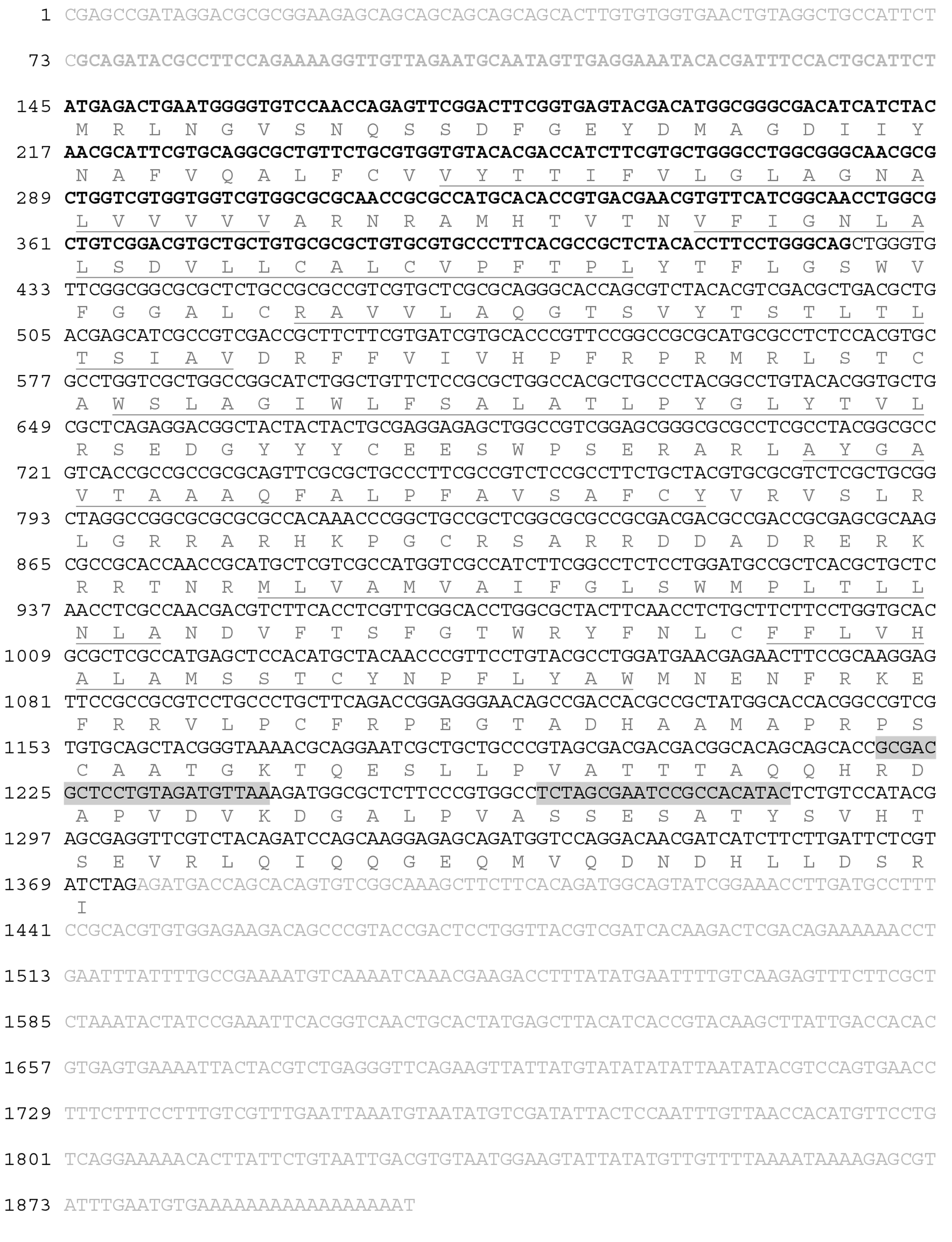

Supplement: Figure S1 — Nucleotide sequence and corresponding amino acid sequence of the short neuropeptide F receptor transcript of Schistocerca gregaria . The open reading frame is printed in black, 5′- and 3′-untranslated regions are printed in grey, sequences predicted as transmembrane segments are underlined, primer sequences used in qRT-PCR assays are highlighted, and the region corresponding to the dsRNA used in RNAi studies is printed in bold. (TIF) [file pone.0053604.s001.tif]

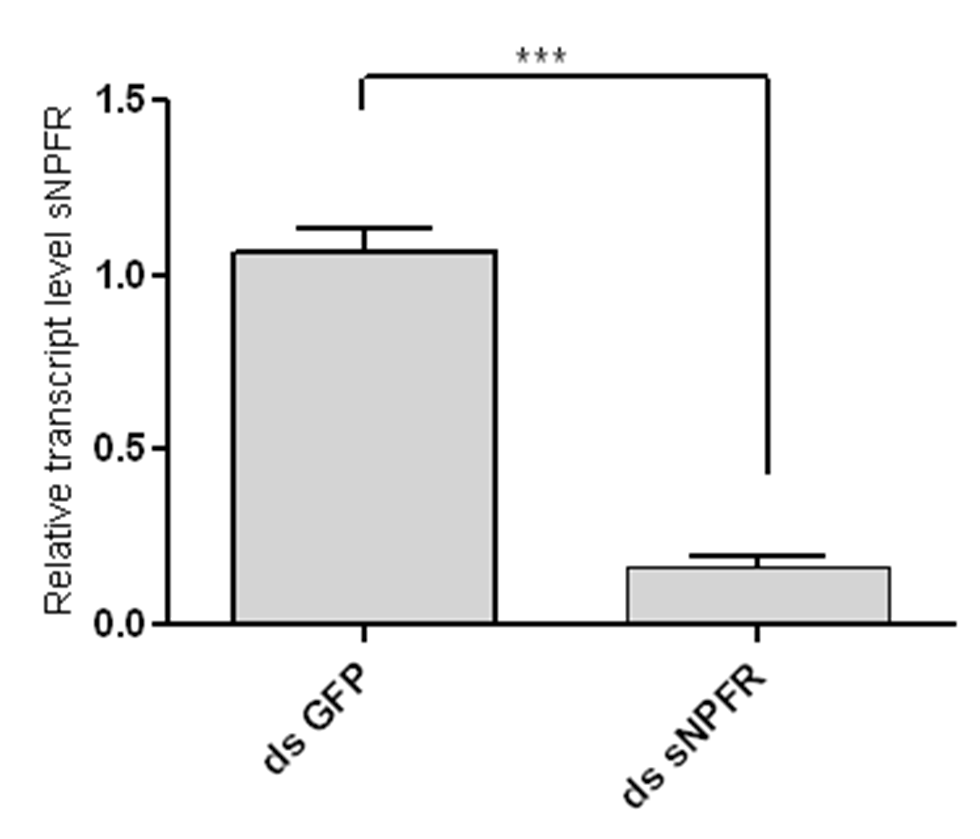

Supplement: Figure S2 — Relative levels of the sNPF receptor transcript upon Schgr- sNPFR knockdown. Locusts were injected with 200 ng dsRNA corresponding to either GFP or sNPFR. Injection of sNPFR dsRNA resulted in a 85% reduction in transcript levels five days after injection. Data represent mean values ± SEM (n = 8). Results were analysed using Student’s t test. *** p<0.001. (TIF) [file pone.0053604.s002.tif]
